# Clinical Management of Mantle Cell Lymphoma With Concurrent Vascular Complications: A Case Report

**DOI:** 10.7759/cureus.57631

**Published:** 2024-04-04

**Authors:** Hemanthkumar Athiraman, Mani Maheshwari

**Affiliations:** 1 Hospital Medicine, Banner Health, Phoenix, USA; 2 Hospital Medicine, Banner Health, Mesa, USA

**Keywords:** inguinal lymphadenopathy, ct (computed tomography) imaging, deep venous thrombosis (dvt), pulmonary emboli, chromosomal translocation, rituximab therapy, fluorescence in situ hybridization (fish), non-hodgkin lymphoma (nhl), mantle cell lymphoma therapies

## Abstract

This is a case of a 70-year-old patient with no past medical history but a significant family history of cancer, who was admitted with acute pulmonary embolism and left lower extremity deep vein thrombosis concerning malignancy. Further investigations revealed mantle cell lymphoma. This case highlights the complex clinical management of patients presenting with concurrent hematological malignancy and vascular complications.

## Introduction

Mantle cell lymphoma (MCL) is a rare type of non-Hodgkin lymphoma, accounting for about 6% of all non-Hodgkin lymphoma cases [1]. It is characterized by aggressive behavior and poor prognosis [2]. Its association with vascular complications such as pulmonary embolism (PE) and deep vein thrombosis (DVT), though rare, presents a complex clinical challenge [3]. This report discusses a case of a 70-year-old patient with MCL presenting with PE and DVT, requiring an intricate, multidisciplinary approach to care.

## Case presentation

A 70-year-old patient with a significant family history of cancer presented with worsening left lower extremity swelling for over a week. The patient reported painful neck and groin lymph nodes and was positive for night sweats but had no weight loss. 

Upon presentation, the patient's vitals were as follows: temperature: 37 °C (oral), heart rate: 89 (peripheral), respiratory rate: 16, blood pressure: 112/70, SpO_2_: 93%, height: 154.9 cm, weight: 66.9 kg, and BMI: 27.88. Laboratory and pathology data is shown in Table 1.

**Table 1:** Laboratory and pathology data BUN: Blood urea nitrogen; GFR: glomerular filtration rate

White Blood Cell Count	22.9 K/uL (normal range is 4-10K/uL)
Red Blood Cell Count	4.24 M/uL (normal range is 3.92 to 5.13 M/uL)
Hemoglobin	11.3 g/dL L (normal range is 12-15 g/dL)
Hematocrit	36.50% (normal range is 36%-47%)
Platelet	57 K/uL (normal range is 150-400 K/uL))
Neutrophils %	24.00% (normal range is 55–70%)
Lymphocytes %	58.00% (normal range is 20–40%)
Monocytes %	1.00% (normal range is 2-8%)
Eosinophils %	0.00% (normal range is 1-4%)
Basophils %	0.00% (normal range is 0.5-1%)
Other cells %	17.00%
Stomatocytes	2+
Platelets	Decreased
Peripheral Smear	Atypical lymphocytes present. No blasts identified.
Prothrombin Time	11.7 seconds (normal range is 11-13.5 seconds)
INR	1 (normal range is 0.8-1.3))
Glucose	104 mg/dL (normal range is 70 to 100 mg/dL)
BUN	13 mg/dL (normal range is 6 to 20 mg/dL)
Creatinine	0.56 mg/dL (normal range is 0.6 to 1.3 mg/dL)
GFR	98 mL/min/1.73 m2 (normal range is 90-120 mL/min/1.73 m2)
BUN/Cr ratio	23 (normal range is 10-20)
Sodium	139 mmol/L (normal range is 135 to 145 mmol/L)
Potassium	4.1 mmol/L (normal range is 3.7 to 5.2 mmol/L)
Chloride	102 mmol/L (normal range is 96 to 106 mmol/L)
CO_2_	22 mmol/L (normal range is 23-29 mmol/L)
Anion Gap	15 (normal range is 4-12)
Calcium	9.3 mg/dL (normal range is 8.5 to 10.2 mg/dL)
Protein, total	7.1 g/dL (normal range is 6.0 to 8.3 g/dL)
Albumin	3.7 g/dL (normal range is 3.4 to 5.4 g/dL)
Alb/Glob ratio	1.1 (normal range is 1-2)
Bilirubin Total	0.7 mg/dL (normal range is 0.1 to 1.2 mg/dL)
Aspartate Aminotransferase	32 U/L (normal range is 8 to 33 U/L)
Alanine Aminotransferase	13 U/L (normal range is 4 to 36 U/L)
Alkaline Phosphatase	184 IU/L H (normal range is 20 to 130 U/L)
FISH Panel	Positive for IGH::CCND1 rearrangement, TP53 deletion and hyperdiploidy.
	Interpretation: Abnormal.
	Fluorescence in-situ hybridization (FISH) was performed on interphase nuclei using probes localized to the CCND1 (11q13), ATM (11q22.3), D12Z3 (12cen), D13S319 (13q14.2), LAMP1 (13q34), IGH (14q32.3) and TP53 (17p13.1) gene regions. Two hundred nuclei were examined, the results demonstrated multiple abnormalities.
	IGH::CCND1 rearrangement was observed in 187/200 (93.5%) of cells scored.
	TP53 deletion was observed in 175/200 (87.5%) of cells scored.
	Hyperdiploidy was observed in all probesets.
	TP53 deletion is associated with a poor response to therapy. Clinicopathologic correlation is advised.
Chromosome Analysis: Bone Marrow	Of the 20 cells examined, 3 exhibited complex abnormalities, including t(11;14)/ IGH::CCND1 rearrangement, rearrangement involving 3p, an unbalanced translocation involving 6q and 6p, monosomy 12, deletion 17p, and a copy of marker chromosome. The remaining 17 cells appeared to be chromosomally normal. IGH::CCND1 rearrangement was also seen from the concurrent FISH testing. These results are consistent with a diagnosis of mantle cell lymphoma in the context of pathology report. Clinical correlation is advised.

**Figure 1:**
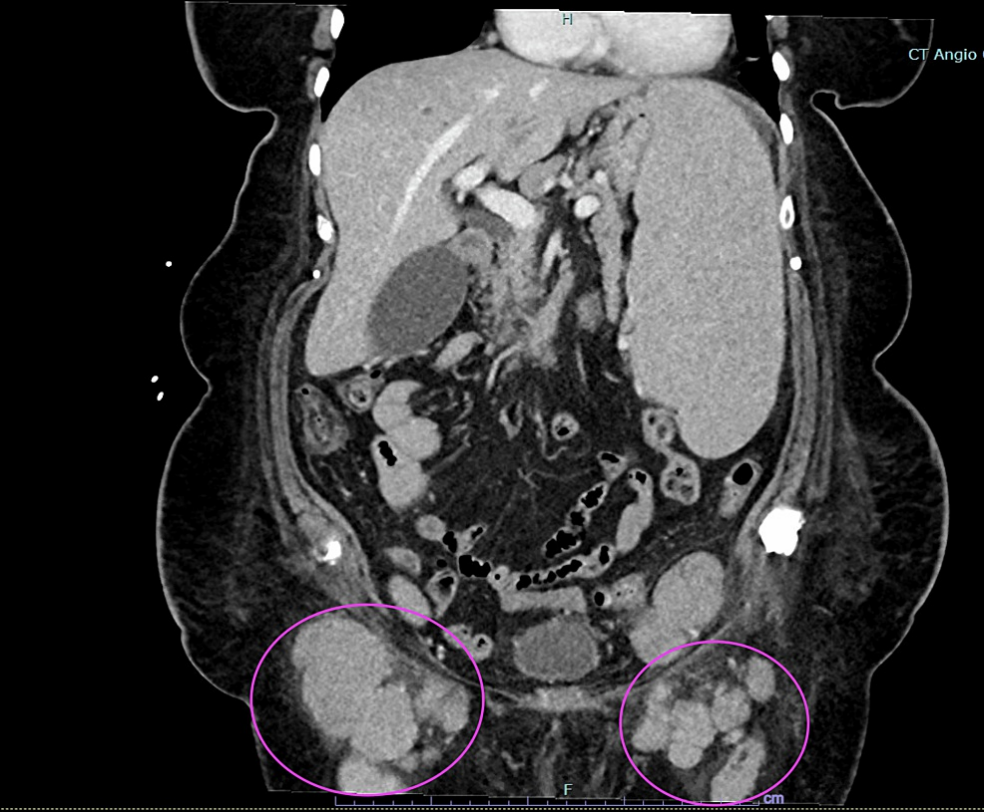
Bilateral inguinal lymphadenopathy on CT imaging (circled in purple color)

Initial CT scans of the chest, abdomen, and pelvis revealed generalized lymphadenopathy in the mediastinum, hila, and axillae; marked splenomegaly with extensive generalized lymphadenopathy; marked extrinsic compression of the left external iliac vein. Marked bilateral groin lymphadenopathy was noted on the physical exam, as seen in the CT scan image (Figure 1). 

Initial workup revealed extensive DVT and PE without any right heart strain. The patient was transferred to a higher level of care, started on intravenous heparin, and underwent a left lower extremity thrombectomy and a repeat thrombectomy 6 days later with IVC filter placement. Unfortunately, given that the patient’s platelet count remained under 50,000, the heparin drip was held intermittently. 

Diffuse lymphadenopathy raised concerns for malignancy, and a biopsy confirmed mantle cell lymphoma. Genetic testing of the patient's cells revealed abnormalities including t(11;14)/IGH::CCND1 rearrangement, rearrangement involving 3p, an unbalanced translocation involving 6q and 6p, monosomy 12, deletion 17p, and a copy of a marker chromosome.

The patient developed acute hypoxic respiratory failure following port placement, necessitating oxygen supplementation. She was concurrently treated for a fungal urinary tract infection and bacterial pneumonia with fluconazole and ceftriaxone. Soon after, Hematology-Oncology started chemotherapy (bendamustine rituximab) during the hospitalization.

Her pancytopenia worsened post-chemotherapy, prompting a blood transfusion. Gradual improvement in pancytopenia was noted, but due to a persistent platelet count of under 50,000, she was discharged without anticoagulation.

Three days post-discharge, she presented to the emergency room with left leg swelling and redness. An extensive left leg DVT, extending from the below-knee veins to the common femoral vein, was diagnosed via venous ultrasound. After consultation with vascular surgery, thrombectomy was performed. Given her improving thrombocytopenia, she was discharged on rivaroxaban. She was also advised to promptly follow up with Hematology-Oncology for further chemotherapy and anticoagulation management.

## Discussion

MCL is an aggressive form of non-Hodgkin lymphoma that typically presents in the later stages of life, with a median age of diagnosis around 68 years [4]. It is characterized by the t(11;14)(q13;q32) chromosomal translocation, resulting in overexpression of cyclin D1, which promotes cell cycle progression and proliferation [5]. The disease is often widespread at the time of diagnosis, with frequent involvement of the bone marrow and gastrointestinal tract [6].

The clinical course of MCL is typically aggressive, with a median overall survival of 3-5 years from diagnosis [7]. However, a small subset of patients may have an indolent course with prolonged survival [8]. Treatment choice depends on the patient's age, performance status, and comorbidities and may include chemotherapy, immunotherapy, targeted therapies, stem cell transplantation, or a combination of these [9]. Genetic abnormalities in the patient's cells, particularly t(11;14)/IGH::CCND1 rearrangement, further complicated the clinical picture, as this genetic abnormality has been associated with aggressive disease and poor prognosis [10].

In this case, the patient's MCL was associated with concurrent PE and DVT, which required careful clinical management to balance the risks and benefits of anticoagulation with the need for cancer treatment [11]. This case underscores the importance of a multidisciplinary care approach involving hematology, oncology, and vascular medicine specialists [12].

Prognostic factors beyond the t(11;14) translocation, the Ki-67 proliferation index, are a marker of cell division rate, and lactate dehydrogenase (LDH) levels, an enzyme found in many body tissues, can also influence MCL prognosis. High Ki-67 and LDH levels are generally associated with a worse prognosis [13].

Treatment considerations should include if the patient received chemotherapy due to the aggressive nature of MCL. R-CHOP (rituximab, cyclophosphamide, doxorubicin, vincristine, and prednisone) is a common chemotherapy regimen for MCL. Newer treatment options include immunomodulatory drugs, Bruton's tyrosine kinase inhibitors, and BCL-2 inhibitors, which have shown promising results in clinical trials [14].

Management of PE/DVT in MCL is a complex process that must consider the patient's risk of bleeding, the need for anticoagulation to prevent further thrombotic events, and the patient's ongoing cancer treatment. Guidelines recommend low-molecular-weight heparin as a first-line treatment for cancer-associated thrombosis, with direct oral anticoagulants as an alternative option [15]. Emerging therapies and research into novel therapeutic strategies for MCL are ongoing. Promising developments include CAR-T cell therapy, which uses a patient's own immune cells to fight cancer, and other targeted therapies that aim to exploit specific vulnerabilities in cancer cells. These emerging therapies offer hope for improved outcomes in MCL [16].

## Conclusions

Patients with MCL often present with widespread disease since there are no obvious symptoms. MCL patients require aggressive treatment, as the literature shows that the first round of therapy has the best chance at remission. A small subset of patients may have an indolent course, and hence, less aggressive treatment strategies are utilized. It is important to note that genetic abnormalities such as t(11;14)/IGH::CCND1 rearrangement should be considered in managing MCL. The literature shows that patients with this rearrangement of genes have poorer responses to chemotherapy. Additionally, patients with the deletion of the p53 gene also have a poorer prognosis. The patient, in this case, had both of these genetic abnormalities, and treatment with rituximab (immunotherapy) and bendamustine (chemotherapy) was started quickly, both of which are primarily used for the treatment of MCL. 

Patients with MCL and concurrent PE/DVT require a careful balance of anticoagulation and cancer treatment. Anticoagulation is essential to prevent further thromboembolic events. However, since almost all chemotherapy agents cause a decrease in blood cells, and at times, significant pancytopenia can occur. Therefore, the setting of anticoagulation poses a marked increase in the risk of bleeding secondary to thrombocytopenia. For optimal patient outcomes, a multidisciplinary approach involving hematology-oncology and vascular medicine specialists is crucial. Meticulous monitoring of the patient’s symptoms, laboratory assessment, and being vigilant for any signs of bleeding/blood loss is crucial.

## References

[1] Romaguer J. E., & Wang M. (2019). Mantle cell lymphoma: a spectrum from indolent to aggressive disease. https://link.springer.com/article/10.1007/s00428-015-1840-6.

[2] Vose JM (2017). Mantle cell lymphoma: 2017 update on diagnosis, risk-stratification, and clinical management. Am J Hematol.

[3] Khorana AA, Francis CW, Culakova E, Kuderer NM, Lyman GH (2007). Thromboembolism is a leading cause of death in cancer patients receiving outpatient chemotherapy. J Thromb Haemost.

[4] Dreyling M, Campo E, Hermine O (2017). Newly diagnosed and relapsed mantle cell lymphoma: ESMO Clinical Practice Guidelines for diagnosis, treatment and follow-up. Ann Oncol.

[5] Swerdlow SH, Campo E, Pileri SA (2016). The 2016 revision of the World Health Organization classification of lymphoid neoplasms. Blood.

[6] Sander B, Quintanilla-Martinez L, Ott G (2016). Mantle cell lymphoma--a spectrum from indolent to aggressive disease. Virchows Arch.

[7] Cheah CY, Seymour JF, Wang ML (2016). Mantle cell lymphoma. J Clin Oncol.

[8] Martin P, Leonard J (2011). Is there a role for "watch and wait" in patients with mantle cell lymphoma?. Semin Hematol.

[9] Ruan J, Martin P, Shah B (2015). Lenalidomide plus rituximab as initial treatment for mantle-cell lymphoma. N Engl J Med.

[10] Beà S, Valdés-Mas R, Navarro A (2013). Landscape of somatic mutations and clonal evolution in mantle cell lymphoma. Proc Natl Acad Sci U S A.

[11] Khorana AA, Kuderer NM, Culakova E, Lyman GH, Francis CW (2008). Development and validation of a predictive model for chemotherapy-associated thrombosis. Blood.

[12] Debourdeau P, Farge D, Beckers M (2013). International clinical practice guidelines for the treatment and prophylaxis of thrombosis associated with central venous catheters in patients with cancer. J Thromb Haemost.

[13] Khorana AA, Dalal M, Lin J, Connolly GC (2013). Incidence and predictors of venous thromboembolism (VTE) among ambulatory high-risk cancer patients undergoing chemotherapy in the United States. Cancer.

[14] Lyman GH, Bohlke K, Khorana AA (2015). Venous thromboembolism prophylaxis and treatment in patients with cancer: American Society of Clinical Oncology Clinical Practice Guideline Update 2014. J Clin Oncol.

[15] Pérez-Galán P, Dreyling M, Wiestner A (2011). Mantle cell lymphoma: biology, pathogenesis, and the molecular basis of treatment in the genomic era. Blood.

[16] Ghielmini M, Zucca E (2009). How I treat mantle cell lymphoma. Blood.

